# Pictorial Phenomena Depicting the Family Climate of Deaf/Hard of Hearing Children and Their Hearing Families

**DOI:** 10.3389/fpsyg.2020.02221

**Published:** 2020-09-23

**Authors:** Anat Avrahami-Winaver, Dafna Regev, Shunit Reiter

**Affiliations:** ^1^Faculty of Education, University of Haifa, Haifa, Israel; ^2^The Emili Sagol Creative Arts Therapies Research Center, The Graduate School of Creative Arts Therapies, University of Haifa, Haifa, Israel

**Keywords:** pictorial phenomena, family squiggle, family climate, deaf/hard of hearing, children

## Abstract

This mixed method study (Explanatory Design – the Participant Selection Model) investigated the use of joint drawing (the Family Squiggle) as a family climate assessment tool for hearing families who have a deaf/hard of hearing (D/HH) child. The goal was to evaluate the possibilities of applying a quantitative approach to characterize the pictorial phenomena produced by hearing families who have a D/HH child and then apply qualitative research approaches to better understand the meaning of these phenomena. Twenty-eight hearing families (parents and child) whose child was diagnosed as D/HH and used hearing devices (hearing aids and implant) were recruited along with 16 families with a hearing child of a similar age enrolled in a mainstream school. The sessions involved a joint drawing followed by a family interview. In the quantitative stage, pictorial phenomena for which there was a significant association between the phenomena and the group of families were defined. These were: (1) the number of dominant images, (2) images occupying less than a quarter of the page, (3) images with accentuated outlines, (4) moderate colorfulness with four to five colors in each drawing, (5) minimal representation of the face, (6) concrete rather than creative titles, (7) muting of conflictual themes, and (8) images reminiscent of hearing devices (hearing aids and implant). In the qualitative phase, interviews were conducted with the hearing families with a D/HH child to better understand the meaning of these pictorial phenomena. The findings suggest that each of these phenomena represent the preoccupation of the family with D/HH, capture a certain aspect of family dynamics, and together provide a broader and deeper picture of the family climate and the interactions between the children’s family and hearing. This assessment tool may thus be utilized when verbal tools cannot be easily applied.

## Introduction

This study applied the Family Squiggle technique as a family climate assessment tool on a sample of hearing families who have a deaf/hard of hearing (D/HH) child. The literature review addresses the emotional characteristics of D/HH children and their hearing families and highlights the complexities of using verbal tools to assess this population. It discusses emerging research in the field of art-based assessment and the potential of the Family Squiggle joint drawing to evaluate family climate.

### Hearing Families Who Have a D/HH Child

Deafness or hearing impairments in infancy and early childhood hinder children’s natural ability for language development and may undermine their emotional, cognitive, and social development (Jackson and Turnbull, 2004; Antia et al., 2009; Marschark and Spencer, 2011). Over 90% of D/HH children are born to hearing parents (Mitchell and Karchmer, 2004; Krieger, 2014). Hearing parents commonly describe their initial response to the diagnosis that their child is D/HH in terms such as grief, trauma, loss, and crisis (Kurtzer-White and Luterman, 2003; Young, 2018). The realization of the child’s condition is often accompanied by feelings of disappointment, denial, guilt, anger, and shame (Ryan-Wenger, 2001; Burger et al., 2005; Lecciso et al., 2013). The family of a D/HH child undergoes a radical change in their ecological environment, which creates a state of imbalance in the family climate. The family dynamics and the development of the child are shaped by the parents’ experience of the loss of the child they hoped and dreamed of (Hardonk et al., 2011; Young, 2018). A decline/loss in hearing has important implications for family interactions and communication, as well as for the child’s sense of security, belonging, identity, and self-worth (Marschark and Spencer, 2011; Macaulay and Ford, 2013). In addition, family dynamics are also influenced by the way the parents perceive the condition of the child, namely, whether deafness is seen as a physiological (hearing) and medical problem to be rectified and “normalized” or as an integral part of the child that relates to a sense of identity and belonging (Marschark and Spencer, 2011; Eichengreen, 2014; Young, 2018). Meadow-Orlans (1990) found that having a D/HH child in a hearing family leads to a more extreme crisis in comparison to a child who is born to a family where one parent is D/HH because the hearing parents’ attitudes are already different during infancy. Other studies have suggested that hearing parents tend to be overprotective and help their D/HH children more with their daily lives compared to their interactions with their hearing siblings (Meadow-Orlans et al., 2003; Mathos and Broussard, 2005).

The literature on the relationship between the D/HH children and their hearing parents (particularly the mother) has focused on the implications of the child’s hearing impairment on interactions with the parents, parental functioning, the choice of communication styles (total communication, sign language, and spoken language), educational attitudes, the child’s personal development, and the family climate (Marschark, 1997; Marschark and Wauters, 2011). The way in which the family deals with hearing loss/deafness depends on a variety of factors related to the child, the parents, and the entire family. Nevertheless, selecting a communication style that is tailored to the child’s needs is crucial for his/her development (Fan, 2012). At the same time, children’s ability to deal with their own D/HH is affected by the way their parents handle the situation. When parents are able to regulate their anxiety, cope better, and establish appropriate relationships with their children, they encourage them to depend on their parents as a reliable source of stability, which in turn provides these children with a sense of security.

For many hearing parents of D/HH children, the discovery of deafness causes tensions in the parent-child relationship leading to feelings of anxiety, inadequacy, disappointment, frustration, and guilt. The child may go through critical developmental stages without being able to communicate and discuss abstract ideas, feelings, and information about the world such as reality and fantasy. As a result, these children often face challenges, such as parental disconnectedness, and emotional distance, difficulties in developing an identity as a person who is D/HH, and a lack of exposure to Deaf culture (Young, 2018). A key factor affecting how parents experience and cope with their child’s deafness relates to their beliefs about being D/HH and early constructions of the meaning of being deaf. The medical approach views the disability as a bodily impairment that needs to be fixed and overcome to be comparable to a hearing person. The integrative-social approach considers deafness to be one of the child’s characteristics, and the Deaf as a group with unique needs such as sign language, which are of equal importance.

Despite the vast number of studies on D/HH children in hearing families, the findings are inconclusive with regard to emotional facets of these children, arising in part from their family climate (Loots and Devise, 2003; Punch and Hyde, 2010). For example, studies have documented greater isolation, emotional distress (Jackson et al., 2008; Antia et al., 2011), and mental disorders among deaf children than in hearing children (Fellinger and Holzinger, 2011), whereas other studies indicate similarities between deaf children and hearing children on measures such as interactions with mothers (Lederberg and Everhart, 1998), peers, and self-esteem (Jerome et al., 2002). Although the diagnosis and assessment of D/HH children captures their abilities and capabilities, as well as their limitations, studies have argued that inappropriate diagnostic tools may account for these conflicting findings. Thus, more research is needed to better understand the complexities involved (Marschark and Spencer, 2011; Marschark and Wauters, 2011).

### Art-Based Assessment

Art-based assessment focuses on pictorial language and not only relates to formal conscious and unconscious characteristics, such as line quality, color, and composition, but also content and symbolic features, including the creator’s behavior and relationship to the artwork (Betensky, 1995). Previous research in the field has attempted to define pictorial phenomena, generate classifications of pictorial patterns, and determine associations between pictorial phenomena and various criteria and theories. Eisenbach et al. (2015) categorized the visual symbols in 379 spontaneous artworks by 10 survivors of childhood trauma, combined with an in-depth, semi-structured interview with each participant. The symbols, which were analyzed according to Jungian theory, described the story of the trauma and possibly played a role in fostering recovery. Cohen-Yatziv et al. (2018) examined the pictorial phenomena depicted in mother-infant relationship drawings by 18 primiparae in their third trimesters who displayed depressive symptoms. Several recurring themes were identified that art therapists could use to better understand the content of these women’s drawings. Another study addressed parent-child relationships (Sheller, 2007) by assessing insecure attachment in children through the Bird’s Nest Drawing (BND) and the Bird’s Nest Sculpture (BNS) tests. The findings showed that using the metaphor of birds in a nest can lead to a better grasp of children’s internal structures in early pre-verbal relationships which affect their later relationships. The authors suggested that art-based tools are a valuable way to approach children’s attachment patterns and their perceptions of their attachment relationships with their parents.

Kwiatkowska et al. (1978) argued that art can provide a more extensive portrayal of family relationships and dynamics. In the mutual space created by the encounter with art, non-verbal expressions of the self, the other, their relationships, and family communication patterns can emerge. Their years of work with families resulted in the development of a structured drawing tool for assessing families that gathers diagnostic information and examines family communication patterns, family interactions, conflicts, and strengths. The development of the tool was based on the definition of distinguishable pictorial characteristics in the drawings of families in which one member had a psychiatric disorder with reference to the literature on groups of various psychiatric disorders. These included the Human Figure Drawing, the Family Drawing, the Personal Squiggle, and the Family Squiggle Drawing.

The Squiggle technique is a squiggle game developed by Winnicott (1971), which is grounded on the natural and universal developmental process in which children use their first “scribble” to communicate with themselves and their environment. Winnicott posited that the game elicits children’s and adults’ creativity that enables discovery, provides meaning, and can be a measure of mental health. In the Squiggle Technique, the participants are asked to engage in a free scribble and then make an image from the scribble of their choice that materializes through spontaneity, creativity, and free associations. This allows for exposure to and connections with unconscious content and conflicts. Kwiatkowska et al. (1978) developed a Family-Based Squiggle Protocol for family assessment. They found that by using the art products, the therapist or researcher could identify considerable similarity in the thinking styles of artworks of family members in which a member had schizophrenia, beyond what was made explicit in conversations with family members.

The present study examined the Family Squiggle drawing as a tool for assessing the family climate in hearing families who have a D/HH child. The purpose of this mixed-methods work was to apply a quantitative method to define the pictorial phenomena that characterize hearing families who have a D/HH child and then apply a qualitative research approach to better understand the meaning of these phenomena.

## Materials and Methods

### Participants

Forty-four families composed of parents and a D/HH child took part in this study. The experimental group consisted of 28 families made up of hearing parents and a child diagnosed with D/HH who uses a hearing device (hearing aids and cochlear implant) enrolled in an educational setting catering to students who have no other disabilities. The control group was composed of 16 families, each with a hearing child enrolled in mainstream education of a similar age to the experimental group. Table 1 lists the demographic characteristics of both groups.

**Table 1:** Child and family demographic variables for the study and control groups.

	Group status	
Variables	Hearing child (*N* = 16)	D/HH child (*N* = 28)
**Age M (SD)**		
Age of child (years)	10.39 (1.10)	10.66 (1.52)
Age of mother (years)	40.93 (3.94)	41.29 (5.77)
Age of father (years)	42.5 (4.20)	42.65 (5.76)
**Gender of child%**		
Male	44%	25%
Female	56%	75%
**Marital status%**		
Married	81%	57%
Divorced	19%	36%
Single parent	0%	7%
**Socioeconomic status%**		
High	56%	14%
Medium	44%	72%
Low	0%	14%
**Education – Average%**		
College	100%	68%
High school	0%	25%
Elementary school	0%	7%
**Degree of hearing loss%**		
Mild (26–40 dB)		18%
Moderate (41–55 dB)		43%
Severe (56–90 dB→)		39%
**Hearing devices%**		
Implant		17%
Hearing aids		75%
Implant + hearing aids		8%
**Communication styles%**		
Spoken language		86%
Sign language		0%
Total (spoken + sign)		14%

The qualitative part of the study was composed of the 28 experimental group families of hearing parents and their child diagnosed as D/HH.

### Research Tools

#### The Family Squiggle

This joint drawing technique was developed by Kwiatkowska et al. (1978). In the warm-up stage, each participant in the family (parents and child) is asked to select one shade of pastel from a set of 48 colors, draw a personal squiggle on a sheet of paper (50 × 70 cm), and then create an image from the squiggle in a variety of colors. The squiggle is made while standing in front of an individual easel (The drawings from the warm-up stage were not analyzed in the present study). In the next stage, the family is asked to draw a squiggle jointly on a separate sheet of paper (100 × 70 cm) with each participant using the original color that they chose and to stop drawing upon instruction, when further drawing would make it difficult to identify individual images. Thereafter, each participant is asked to observe the squiggle drawing that they made together, find images, and then tell the other participants what images they found. The family then selects one of these images, marks it, and makes a drawing based on this image using the full palette of pastels. The family is told that they can add details to the image or disregard parts of the scribble they feel they do not need. Finally, the family decides on a title for the artwork. In the current study, the parents and the child worked on the joint squiggle together at one easel so that they could move around. All decisions about the drawing were made by the family members together and also reflected the relationships within the family.

To document the pictorial phenomena in the joint drawings, a phenomenological content analysis was conducted on the drawings of 10 families. This included observable phenomena in the art products, such as image size, position on the page, number of images, and line or color accentuation (Betensky, 1995). The purpose was to identify specific phenomena potentially applicable to a richer understanding of the family climate of each family. The phenomenological content analysis was conducted by two teams: the first author of this article took part in both teams in addition to another art therapist on each team, each of whom has extensive experience in the field and work as supervisors and lecturers at the School of Creative Arts Therapies at the University of Haifa. The teams discussed the relevance of each phenomenon to family climate until consensus was reached.

In the second stage, to test reliability of the tool, three experienced art therapists analyzed three identical drawings independently. They used the phenomena defined in the previous stage and were requested to decide whether they were present or absent in the three drawings. The degree of agreement was calculated to assess reliability of the tool. The Kappa reliability score ranged from 0.58 to 0.97. Since the reliability score was good, all 44 family drawings were then analyzed by the first author and two other art therapists who teach at the University of Haifa.

#### Semi-Structured Interview

At the end of the Squiggle session, a semi-structured interview was conducted with the experimental group families composed of hearing parents and a child diagnosed as D/HH, which included talking about the drawing process, a joint observation of the art product and its characteristics, telling a story about the drawing, the family’s perception of the D/HH child, and insights related to the family climate and dynamics. The questions had to do with the experience of each family member, who, for example were asked “What do you see in the drawing?,” and “Describe who you are in the drawing and where you are situated,” as well as shared experiences and dynamics: “Which image did you chose and how does this image feel within the family?,” “Who chose the title for the artwork? Why did you choose it?,” “Is there anything in the story or in the drawing that reminds you of your family?,” and “Is there anything in the image that reminds you of D/HH?.” The interviews were tape recorded and then transcribed to ensure the accuracy of data collection (Creswell, 2014). The interview was based on the phenomenological approach to art therapy, which was originally aimed at increasing the client’s awareness of the experience and the content manifested in the artwork (Betensky, 1995). According to Wadeson (1987), the creator is the most reliable source for completing and reaching conclusions about the information that emerges from the art product. This type of interview can provide information about the creators’ experiences and interpretations of phenomena identified using the phenomenological observation process (Malchiodi, 1998), and provides a broader impression of the family’s climate and attitudes toward the child’s D/HH (Kwiatkowska et al., 1978; Sheridan, 2001).

### Procedure

The families were recruited at schools and centers attended by D/HH children after receiving authorization from the Ministry of Education and the respective schools. Counselors and teachers in the schools agreed to suggest suitable D/HH children and hearing children. They then submitted requests to the children’s parents to participate in the study. The first author contacted the parents who gave their consent and described the study to them.

The meetings with the families took place in their homes and consisted of the art-based assessment and an interview. At the start of the meeting in the homes of families with a D/HH child, the researcher offered to use total communication that involved spoken and sign language at the same time to ensure that the D/HH child would be a full partner in the process and understood everything that was being said. Most families chose to use spoken language alone. For all families, the researcher explained the objectives of the study, its relevance, and how it would be conducted. The families were also told that the interview would be tape recorded and that their drawing abilities or talents were not being examined. The family members made an initial personal squiggle for purposes of familiarization with the technique before the joint squiggle drawing and family interview while observing the artwork.

### Ethics

Prior to engaging in the squiggle drawings, the participants were informed that they were taking part in a study on the family climate of hearing families with D/HH children. Families with hearing children were told that they comprised the control group. They were presented with the research procedure, which included the personal drawing (the warm-up), the joint drawing, and the interview. They were told that they could withdraw from the study at any time, that participation was anonymous, and that their confidentiality was guaranteed. Parents were asked to sign a consent form for participation in the study. They were also requested to indicate their permission or refusal to authorize publication of the artworks devoid of any identifiers. The parents were also informed that the study had been approved by the Chief Scientist of the Ministry of Education and that if they were interested, they would be able to obtain a summary of the research upon completion. In addition, given that fact that D/HH children’s understanding and needs are different from those of their parents, several specific steps were taken to address them particularly. The explanation was translated into sign language where applicable to be sure that the child understood what was taking place. The children were also told that they could withdraw from the study at any time.

### Data Analysis

The data processing was carried out using the Explanatory Design – The Participant Selection Model (Creswell and Clark, 2017). The quantitative analysis was conducted first. In order to examine the associations between the pictorial phenomena and the group, chi-square (*χ*^2^) analyses were conducted. In the second phase, the artworks and interviews with the 28 families, in which there were hearing parents and a D/HH child, were analyzed qualitatively. The objective of the interview was to gain a better understanding of the background of the artwork and to serve as an additional source to interpret the meaning of the pictorial phenomena that appeared in the drawings. Transcripts were prepared from the audio recordings and interview notes. After defining the pictorial phenomena, the first author read the transcripts iteratively in an attempt to find references and statements explaining the meaning of the pictorial phenomena to the creator (Eisenbach et al., 2015).

## Findings and Discussion

The pictorial phenomena manifested in the joint drawings of the group of families with a D/HH child were compared to the pictorial phenomena that emerged in the drawings of the group of families with a hearing child. For this purpose, the prevalence of each pictorial phenomenon in each group was calculated and a χ^2^ analysis was conducted to examine associations between the pictorial phenomena and the group. The findings appear in Table 2.

**Table 2:** *χ*^2^ associations between the pictorial phenomena and the groups of families.

Variable	Frequency		*χ*^2^	Cramer’s V
	D/HH child	Hearing child		
**Organization of the drawing on the page**				
One image dominates in the drawing	86.7	87.5	0.01	
Number of images that dominate in the drawing	20.0	0.0	3.68^+^	0.28
Size of the image occupies more than half the page	46.7	68.8	2.05	
Size of the image occupies less than a quarter of the page	56.7	25.0	4.22^*^	0.30
The image is located in the center of the page	68.8	46.7	2.05	
Elements outside of the image (in the background)	46.7	50.0	0.05	
**Line**				
Continuous outline of the image	70.0	62.5	0.27	
Fragmented lines in the image	23.3	37.5	1.03	
Dominant line	40.0	50.0	0.43	
The outline of an image is accentuated	66.7	37.5	3.61^+^	0.28
**Color marks**				
A dominant color in the image	53.3	56.3	0.04	
Rich color use – 6 or more colors in each drawing	23.3	68.8	9.04^**^	0.44
Moderate colorfulness – 4–5 colors in each drawing	50.0	12.5	6.30^*^	0.37
Poor color use – 3 or fewer colors in each drawing	26.7	18.8	0.36	
**Content**				
Face	20.0	56.3	6.24^*^	0.37
Animals	40.0	31.3	0.34	
Still life – objects	26.7	12.5	1.23	
**Title**				
Compatibility between the title and artwork	100.0	93.8	1.92	
Concrete title	66.7	25.0	7.26^**^	0.40
Creative title	26.7	81.3	12.53^**^	0.52
**Themes**				
Conflict (teeth, pitchfork, Ninja, and aggressive expression)	13.3	37.5	3.58^+^	0.28
Addition of details to the head of an image	40.0	31.3	0.34	
Hearing aids (device and implant)	50.0	25.0	2.69^+^	0.24
Image in the shape of an ear, accentuation or prominence of the ears	33.3	18.8	1.10	

+ *p* < 0.1

* *p* < 0.05

** *p* < 0.01.

The following section refers to the pictorial phenomena that were significantly associated with the groups of families. These phenomena corresponded to meaningful images related to the D/HH child’s world and his/her family climate as they emerged from the interviews with the families. All names are fictitious and only drawings for which parents gave their approval are reproduced.

### The Number of Dominant Images

**Figure 1:**
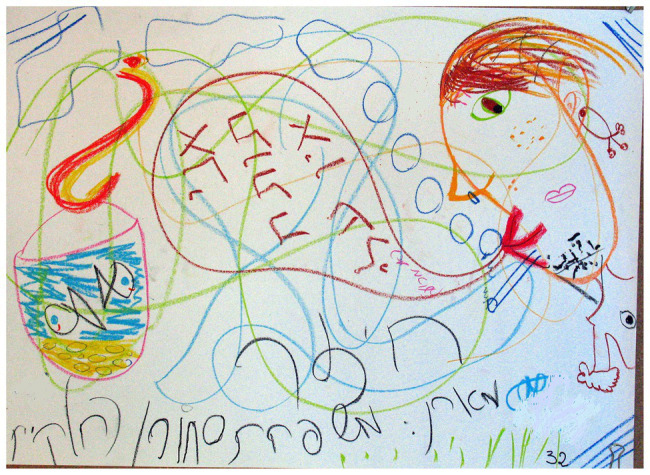
Several images dominate in the drawing.

**Figure 2:**
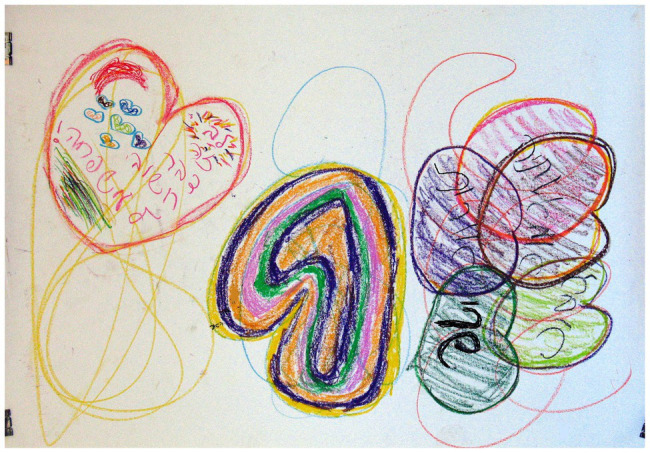
Several images dominate in the drawing.

A marginally significant association was found between “The number of dominant images” and the two groups of families (*χ*^2^ = 3.68, *p* < 0.10). As shown in Table 2, in families with a D/HH child, there was a higher percentage (20.0%) of the use of this phenomenon than in families with a hearing child (0.0%). This phenomenon relates to the participants’ choice to draw more than one image despite the fact that they were instructed to select and draw only one (see Figures 1, 2). The following example illustrates one family’s reference to multiple images: “Einav chose Mother’s fish. Father said: if these are Mother’s fish then there will be both fish and my drawing of the question mark… In the end everyone got what they wanted: a question mark, a fish and a head.” In this example, as well as in others, the multiple images seemed to be related to issues of relationships between family members represented through their images. On the one hand, the need for each member of the family to want to include his or her own image may indicate a willingness to accept a variety of images and suggests an attempt to provide space for each member of the family (Minuchin, 1974). There is evidence in the literature that the presence of a disability in the family can strengthen family ties. For example, Zaidman-Zait et al. (2020) found that siblings of children with intellectual disabilities displayed closer relationships, commitment, and tremendous support for siblings and family. However, this could also indicate the prevalence of complex relationships, such as difficulties in forming cohesion, inflexibility, or competitiveness, where family members cannot relinquish their personal images and fail to settle on a single family image.

### Images Occupying Less Than a Quarter of the Page

**Figure 3:**
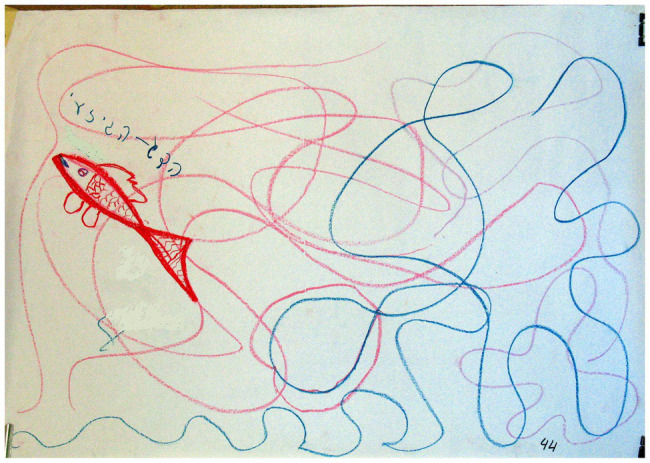
The image occupies less than a quarter of the page.

**Figure 4:**
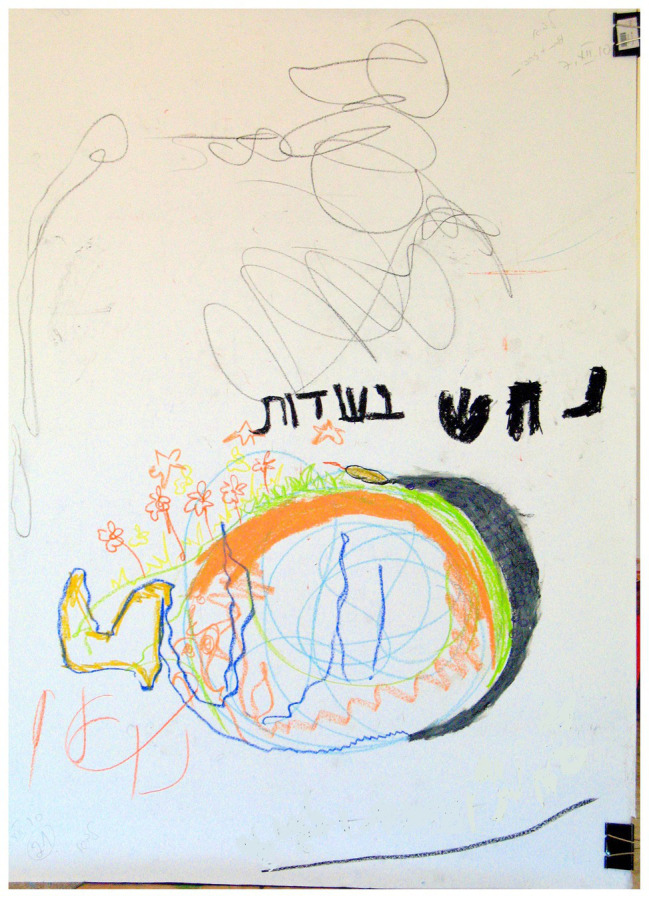
The image occupies less than a quarter of the page.

A significant association was found between “Images occupying less than a quarter of the page” and the two groups of families (*χ*^2^ = 4.22, *p* < 0.05). As shown in Table 2, in families with a D/HH child, there was a higher percentage (56.7%) of the use of this phenomenon than in families with a hearing child (25.0%); for example, see Figures 3, 4. In the literature, small images have been linked to the perception of a small self, low self-esteem, and the experience of diminution and insecurity in relation to the self and the environment (e.g., Di Leo, 1973; Dolidze et al., 2013). Given that this was an image in a family drawing, it can be interpreted in several ways. If the child chose the size of the image, it may reflect low self-esteem that the child attributes to his/her D/HH. The participants themselves referred to the image that appeared in the drawing as a small and dependent object. This was manifested in a verbal exchange between a mother and her child: “Mother: Mother fed her little snake Crotalus… Son: He could not walk… Mother: And they had fun crawling together.” It is assumed that a child’s low self-esteem materializes as a result of communication difficulties, the experience of being different from others, overprotective parents who continue to relate to their child as a baby, and the child’s dependence on his/her parents (Desselle, 1994; Dolidze et al., 2013). If, however, the image was the outcome of interactions among family members, it could convey the family’s experience of being different, thus reflecting the family’s experience of being part of a minority group, much like the experience of the child in his/her individual environment. Therefore, the familial experience appeared to derive from the interconnectedness between the child’s experience and the parents’ experience (Zaidman-Zait, 2008; Antonopoulou et al., 2012). Finally, a small family image size could also express the family’s need to conceal or deny the D/HH condition by reducing and minimizing its presence, possibly reflecting the wish that the deficiency would not be seen and therefore not felt (Eichengreen, 2014).

### Images With Accentuated Outlines

**Figure 5:**
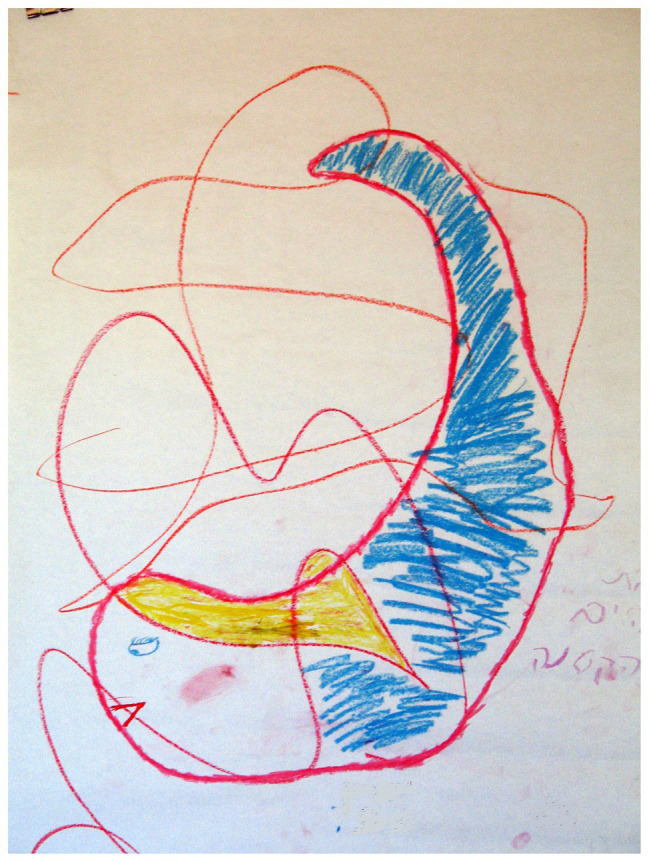
Accentuated of the image – heavy pressure on the pastel stick.

**Figure 6:**
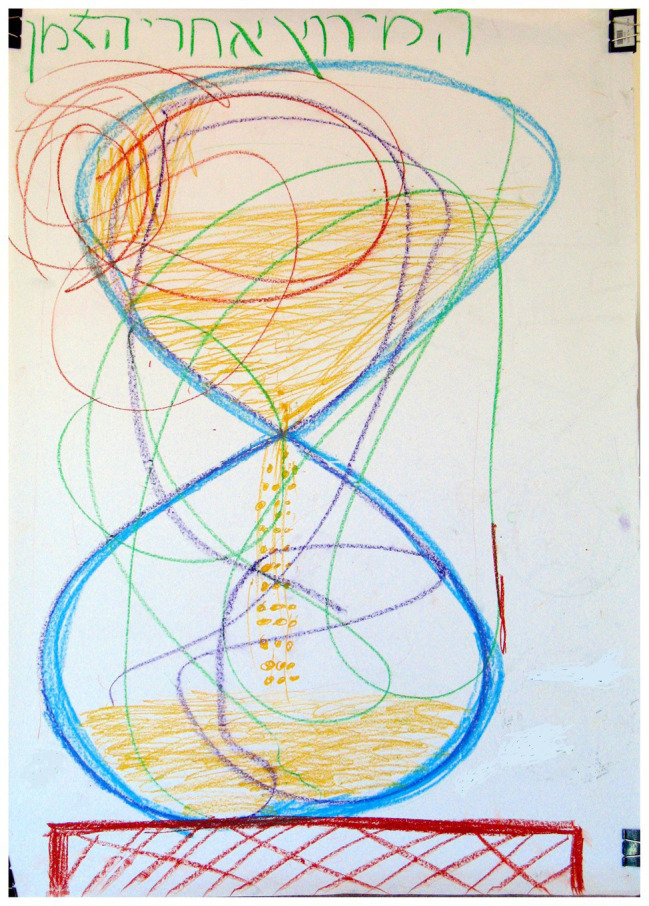
Accentuated outline of the image – redrawing over the lines.

A marginally significant association was found between “Images with accentuated outlines” and the two groups of families (*χ*^2^ = 3.61, *p* < 0.10). As shown in Table 2, in families with a D/HH child, there was a higher percentage (66.7%) of the use of this phenomenon than in families with a hearing child (37.5%). This pictorial phenomenon was characterized by heavy pressure with the pastel sticks (see Figure 5) or by overlays of lines (see Figure 6). Participants referred to the accentuated lines as an expression of the need to maintain control and organization during the creative work, as seen in the comments and behavior of one of the mothers about an hourglass drawn by the family: “Father thinks you should make the top section larger and bolder… Let me, I will draw it.” The accentuated and controlled line in the hourglass and its title were confirmed later in her remarks: “Specifically the hourglass fits… For me everything has to be exact.” The literature on the analysis and understanding of pictorial phenomena considers an accentuated line as an expression of control, stress and anxiety, rigidity, and limitations, which creates a type of divide between the creator and his/her environment and may reflect a need for protection and separation between the outer and inner worlds (Lev-Wiesel and Yosipov-Kaziav, 2005; LaRoque and Obrzut, 2006). Control and organization may provide parents with a sense of security in a situation that is often fraught with anxiety and concern (Krieger, 2014). For example, most hearing parents tend to communicate verbally with their D/HH child. Parents expect the child to communicate in “normal language,” which means they will communicate like them, and they take steps such as integrating the child into the mainstream school systems so that the child acquires language and functions similar to hearing children and the hearing culture (Young, 2018). Studies examining parental experiences and responses to receiving diagnoses of their child’s illness or disability indicate that parents attempt to deal with the problem by controlling and attempting to change or normalize the situation (Patterson and McCubbin, 1983; Young, 2018). Lev-Wiesel and Zeevi (2007) found more barriers and splits in mother and child drawings of mothers of children with Down syndrome compared to a group of children without disabilities. They suggested that there may be difficulties in communication, negative feelings about the child, and an avoidance of dealing with stress as a result of parenting a child with Down syndrome.

There are several other explanations for these families’ need to accentuate the outlines of their images. A bold continuous line creates a type of a shield and may serve as an expression of detachment and a sense of being different and strange that are associated with a D/HH children’s experience (Young, 2018). These children experience a sense of being different, detachment, and an absence of belonging (Jackson et al., 2008), and the family also experiences itself as different from families with hearing children (Horovitz, 2007). Alternatively, the accentuated outline of the family’s drawing can be seen as expressing the needs of the child and the family for visibility, when their voices are not sufficiently heard. It is possible that the accentuated visual components functioned to proclaim and validate the D/HH condition within the family. The parental experience may be one of feeling different (Henderson and Hendershrtt, 1991), combined with the feeling that their voices are not heard by official actors, such as educational frameworks.

### Moderate Colorfulness With Four to Five Colors in Each Drawing

**Figure 7:**
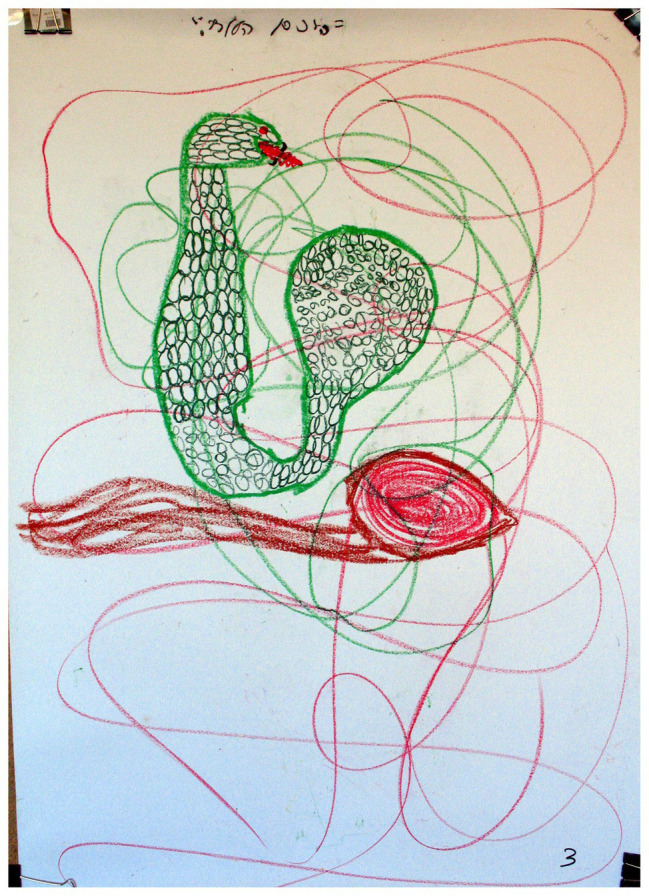
Moderate colorfulness – 4–5 colors in each drawing.

A significant association was found between “Moderate colorfulness with four to five colors in each drawing” and the two groups of families (*χ*^2^ = 6.30, *p* < 0.05). As shown in Table 2, in families with a D/HH child, there was a higher percentage (50.0%) of this phenomenon than in families with a hearing child (12.5%). This phenomenon was characterized by the use of four to five colors in the drawings (see Figure 7) by families with a D/HH child in comparison to the use of six colors or more in drawings by families with a hearing child. Colorfulness in drawing is attributed to emotional and thoughtful expression, flexibility, and creativity (Betensky, 1995; Gantt and Tabone, 1998; Winaver-Avrahami, 2001). It is, therefore, possible that for this particular phenomenon, the lesser use of color in the drawing by families with D/HH children may have been related to the issue of family communication (Krieger, 2014), and what Horovitz (2007) terms “the family myth” of families with D/HH children, where the child may be viewed as less intelligent and more helpless. This myth can affect the family’s emotional expression, flexibility, and level of creativity. However, according to Horovitz (2007), a child’s poor and limited communication skills, which are expressed in restricted language and communication, and the implications this has for learning processes, do not necessarily indicate lower potential. Krieger (2014) found that D/HH children whose families communicated in sign language used a variety of colors in the Kinetic Family Drawing task and presented normative emotional and cognitive development. In the present study, the extent of colorfulness that characterized the family drawings with D/HH children was halfway between the poor and rich colorfulness that characterized the color use in family drawings with hearing children. The moderate range of colors may indicate the potential for the development and growth of these children. The Squiggle joint drawing may have helped D/HH children with self-expression but it is also likely that good communication could also enhance the richness of their verbal language. This emerged in families who used verbal communication and had good relationships, as evidenced in their ability to show empathy, parental mediation, and high levels of mentalization. This was crystalized in the example below of the whale story (which included five colors) in a family who communicated using verbal language:

Daughter: The whale had no colors, when he was white he wanted to change and be like all the other whales. Father: He turned and cried and he met a very clever seagull who asked him: “Why are you crying?” Daughter: Then the whale said he was crying because he had no colors, he really wanted to be colorful and everyone laughed at him because he was all white and everyone had different colors. Mother: Then the seagull said to him: “Do you want to be colorful? I can arrange for you to be colorful.” And he flew ashore. Daughter: He flew to the beach which was full of people wearing colorful swimsuits, and he took their clothes and then he went back to the whale and said: “Here you go, I have lots of colorful swimsuits for you. Take their colors.” Father: And then the whale swam away and showed off his colors to all the fish: “Look how colorful I am. And look how beautiful I am.”

### Minimal Representation of the Face

**Figure 8:**
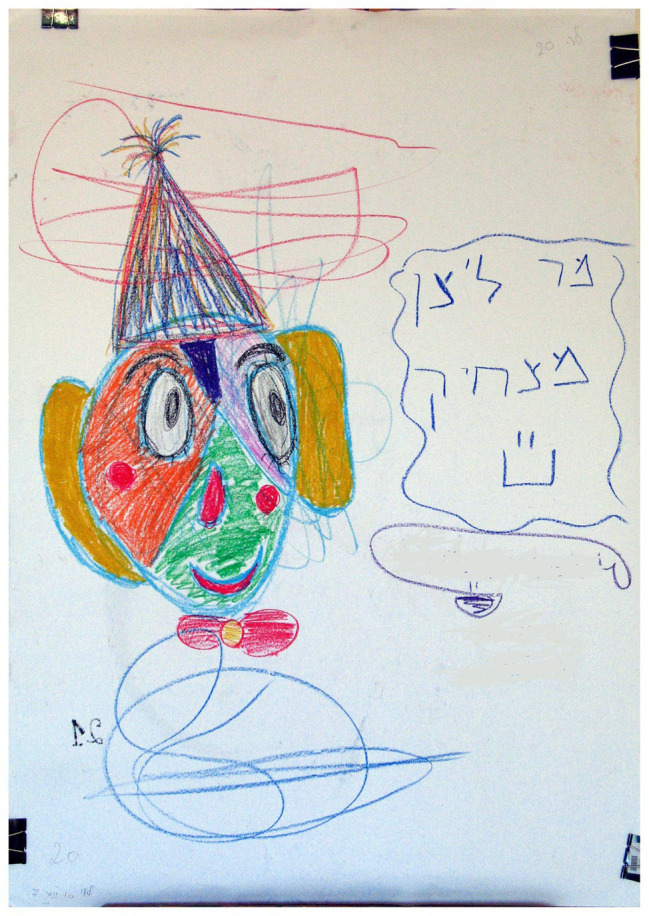
Image of a face.

A significant association was found between “Minimal representation of the face” and the two groups of families (*χ*^2^ = 6.24, *p* < 0.05). As shown in Table 2, in families with a D/HH child there was a lower percentage (20.0%) of this phenomenon than in families with a hearing child (56.3%). The face, as part of the body image, appeared less frequently in the drawings by families with D/HH children compared to the presence of faces that appeared more frequently in the drawings by families with a hearing child. The assumption is that drawings of the face are associated with a position of emotional openness and confidence, creativity, and the ability to deal more effectively and positively with difficulties and conflicts such as direct and authentic encounters with the self and self-identity of each individual in the family. By contrast, the minimal presence of the face in drawings may be an expression of unconscious and layered projections that reflect a difficulty in dealing with the self and possibly implies a preoccupation with D/HH issues, such as parental reference to deafness, and issues relating to the relationship, communication, and conflict. According to Furth (2002), the lack of body images in drawings may indicate the presence of conflict. In addition, the minimal presence of the face in images also involved a limited presence of the ears, the impaired hearing organ of the face. However, an examination of the family drawings in which the face did appear showed that the ears or eyes were visually emphasized (see Figure 8), which may reflect the impairment of the sense of hearing and the need to employ other senses, such as sight, touch, smell, and taste, to deal with the external world (Lev-Wiesel and Yosipov-Kaziav, 2005; Hidayat et al., 2017). Studies have also shown that when deaf people were asked to draw a human figure they drew a figure with either emphasized or completely absent ears (Lev-Wiesel and Yosipov-Kaziav, 2005).

**Chart 1:**
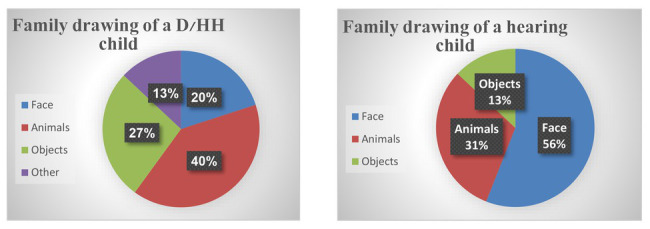
Proportion of faces, animals and objects in the two groups. “Other” in the chart refers to a mushroom, a mermaid, hearts, and a moon.

To illustrate the accentuated content in the drawings of families with D/HH children, Chart 1 shows the frequency distribution of the content that appeared in the family drawings. The small number of faces in the family drawings with a D/HH child (20% as compared to 56% in families with hearing children) was compensated for by images of animals, which appeared in 40% of the family drawings with a D/HH child, as compared to 31% in the family drawings with a hearing child.

**Figure 9:**
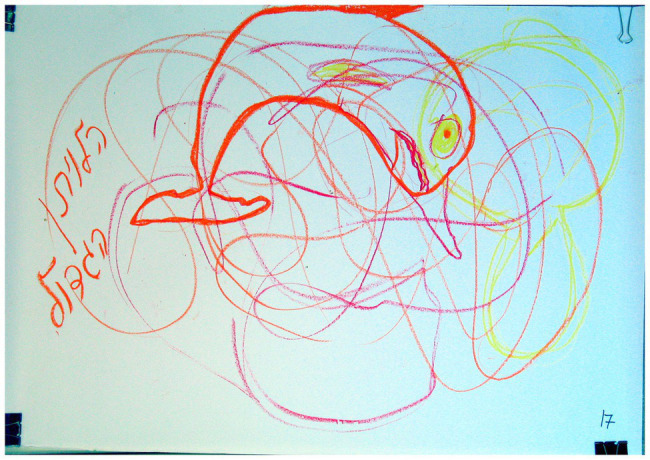
Image of an animal.

These animals included fish, whales, sharks, swans, ducks, butterflies, and snakes. It is important to note that there was no significant association between the animal content and the two groups of families. At the same time, the gap between the groups seems meaningful. In various cultures and myths, societies tend to personify animals and attribute different character traits to them that reflect parts of the person, some of which the individual would like to adopt, or alternatively, renounce (Soto, 2017). The drawings of animals by families with D/HH children included many aquatic animals, including fish, whales, sharks, and ducks (see Figure 9). These animals are devoid of ears, perceive the environment by using other senses, and communicate in a specific language. These images are likely to indicate how the child/family perceives the D/HH condition. The animal category also included terrestrial animals that lack hearing organs such as the snake, which engages with the environment through other senses, much like how the D/HH child perceives the environment. However, the snake also symbolizes the power of healing, transformation, and renewal through skin shedding and replacement (Jung, 1953; Chevalier and Gheerbrant, 1969; Cirlot, 2002).

**Figure 10:**
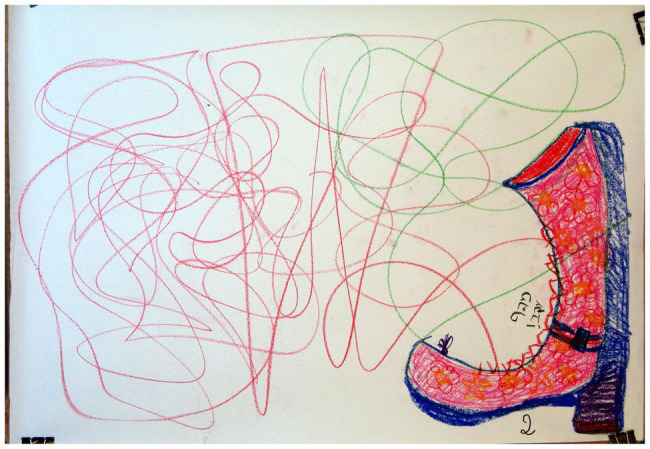
Image of an object.

In addition, images of objects appeared in 27% of the drawings by families with a D/HH child as opposed to 13% of families with a hearing child. It is important to note that there was no significant association between object content and the two groups of families. At the same time, the disparity between the groups seems worth exploring. These images of objects were related to the D/HH child’s world and experiences, such as the tendency for concretization, the issue of communication, or medical procedures in the lives of D/HH children. For example, in the shoe drawing (Figure 10), the family mentioned that the shoe is reminiscent of the child’s hearing aid. It has been claimed that images of objects are a manifestation of language delay and the concrete perception of the D/HH child (Antia et al., 2009; Marschark and Spencer, 2011). This was also expressed in the concrete titles of the artworks (see next section).

### Concrete Rather Than Creative Titles

**Figure 11:**
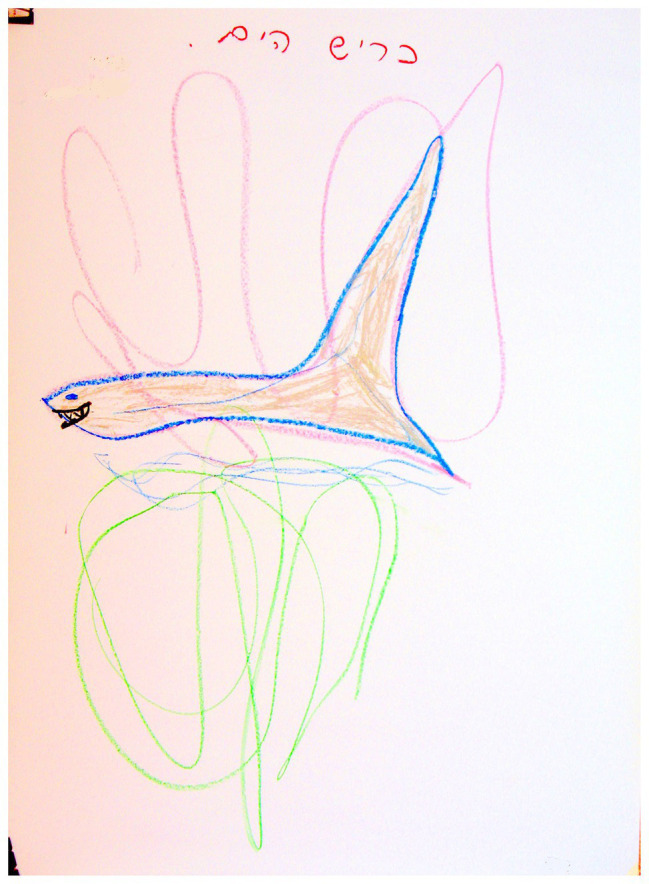
A concrete title – “The sea shark.”

A significant association was found between “Concrete rather than creative titles” and the two groups of families (*χ*^2^ = 7.26, *p* < 0.01). As shown in Table 2, in families with a D/HH child, there was a higher percentage (66.7%) of this phenomenon than in families with a hearing child (25.0%).This phenomenon refers to the title given by the family to the artwork which described the drawing in a tangible and real way (see Figure 11), such as the “The Sea Shark” or “The Cute Fish,” unlike a creative title, which describes the drawing in a creative and abstract way, such as “The Confirmation of a Young Priest.” The title is defined as part of the artwork and allows for the assessment of creative and cognitive abilities (Malchiodi, 1998). It is possible that a concrete title for a family drawing may indicate concrete thinking and restricted creativity that depends on a number of factors, such as poor language use by the D/HH child, a parental communication style that does not include sign language and therefore is not adapted to the child’s needs (Krieger, 2014), a delay in Theory of Mind (Jones et al., 2015), parents’ use of nicknames that exclusively belong to the child’s world, and/or less availability on the part of the parents for enrichment and growth (Horovitz, 2007; Zaidman-Zait, 2008).

### Muting of Conflictual Themes

**Figure 12:**
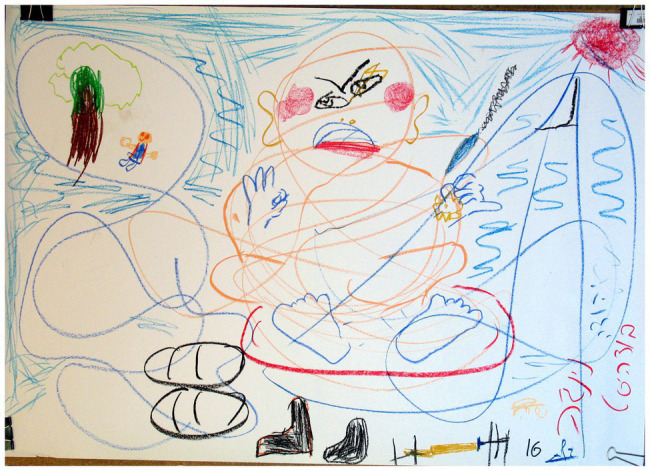
Images with conflicting content.

A marginally significant association was found between “The muting of conflictual themes” and the two groups of families (*χ*^2^ = 3.54, *p* < 0.10). As shown in Table 2, in families with a D/HH child, there was a lower percentage (13.3%) of this phenomenon than in families with a hearing child (37.5%). This theme can be represented by angular and jagged lines, the use of bold color and the distance between images (Betensky, 1995; Malchiodi, 1998; Ferro, 2003), as well as by symbols, such as teeth, sharp accessories, aggressive expressions, and the presence of superhero/villain characters (Horovitz, 2007; see Figure 12). The low prevalence of conflict in the family drawings may be associated with the families’ failure to deal with emotions, such as pain, grief, the experience of being different (Lev-Wiesel and Zeevi, 2007), and the desire to “normalize” the D/HH child (Eichengreen, 2014). This can be seen in the following example: “She is a normal girl, she is treated normally, like a normal child, she has no privileges… Do not relate to the fact that we have a…. a…. girl.” However, the rare references to conflict in the drawings by families with D/HH children may also be associated with the nature of the parent-child relationship characterized by an abundance of empathy, overprotection, and parental support for the child and may also be connected to the child’s level of dependency on his/her parents (Meadow-Orlans et al., 2003; Lev-Wiesel and Yosipov-Kaziav, 2005). This situation does not leave much room for conflict.

### Images Reminiscent of Hearing Devices (Hearing Aids and Implant)

**Figure 13:**
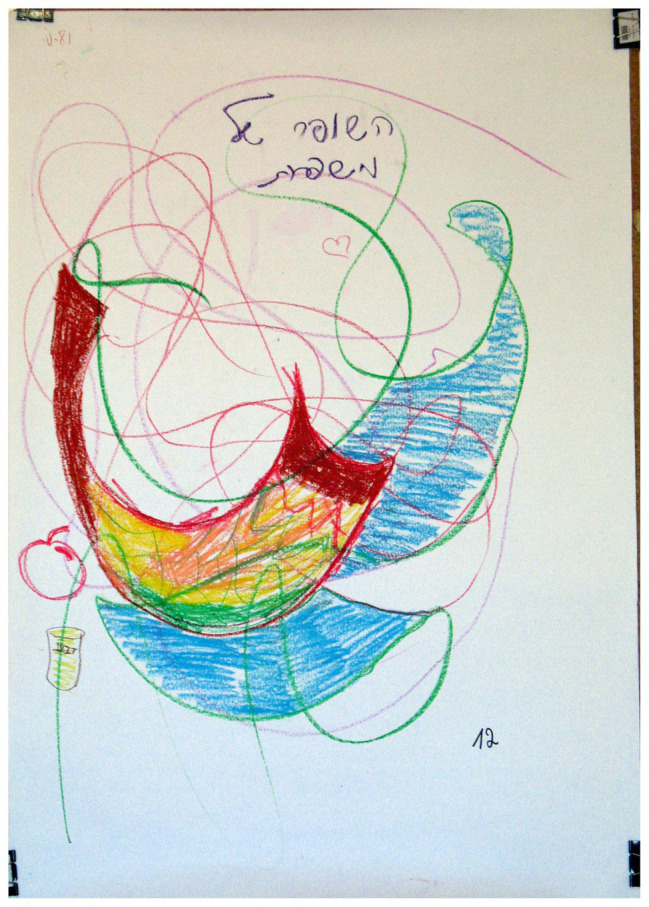
Image of a hearing aid.

A marginally significant association was found between “Images reminiscent of hearing devices” and the two groups of families (*χ*^2^ = 2.69, *p* < 0.10). As shown in Table 2, in families with a D/HH child, there was a higher percentage (50.0%) of this phenomenon than in families with a hearing child (25.0%).This phenomenon refers to images in drawings that looked like or were reminiscent of a hearing device (for a hearing aid, see Figure 13). Most of the participants indicated a connection between this pictorial phenomenon and the hearing aid as presented in the following example:

Daughter: If I look at it like this… It looks like there is a kind of an ear. Father: I see a hearing aid, without a doubt, it is a hearing aid. Mother: Well, now I see, without a headset, the device itself. Father: With the button… Mother: It is the battery, I do not know, it is probably something in our subconscious minds. Father: It comes from our scribble, I’m reminding you. Mother: The scribble, but that is part of our life. Father: We said “scribble” but that is what we saw in the scribble. We could have seen many other things.

Half of the families who depicted the pictorial phenomenon of a hearing aid or implants in their drawings stated that the image reminded them of a hearing device. It is assumed that the drawings of hearing devices that serve as primary means of communication are likely to be loaded with unconscious sensory and emotional meanings which reflect the feelings of the child and the family, such as dealing with the D/HH condition, the experience of being different, emotional communicative disconnection, and issues of identity (e.g., Kwiatkowska et al., 1978; Betts, 2006; Krieger, 2014). Previous research indicates that there is a relationship between pictorial phenomena, such as an image in the form of a hearing devices or accentuation of ear and eye images, and physical problems that are expressed symbolically, structurally, and colorfully (e.g., Malchiodi, 1998; Winaver-Avrahami, 2001; Lev-Wiesel and Yosipov-Kaziav, 2005).

## Summary and Conclusion

There is growing recognition that assessment tools in art therapy can shed light on mental states and processes. In the shared space enabled by the encounter with art, non-verbal perceptions of the self, others, and their relationships and patterns of communication in the family can be expressed. The quantitative part of the present study defined the pictorial phenomena that characterize hearing families who have a D/HH child. The qualitative part then examined the meaning of these phenomena. This summary section describes the conscious and less conscious aspects of the lives of hearing families with D/HH children emerging from the interviews and the pictorial phenomena.

The findings suggested that several pictorial phenomena may reflect the hidden layers of these families’ ways of dealing with a D/HH child. These included images in the form of hearing devices which were created from the joint family images and sincerely surprised the members of the families themselves. Other phenomena, such as the minimal presence of the face and ears, subconsciously helped the parents represent the child with D/HH within the family and sustain their culture, values, and characteristics while nurturing those of their D/HH children.

Several characteristics related to the families’ feelings about the D/HH child emerged. The child’s and family’s struggle to deal with their situation and their unique identity emerged in the form of both small images and the failure to draw faces. A sense of being different, withdrawal, and a sense of otherness of the child and the family were manifested by the presence of small images and their accentuated outlines in an attempt to describe their detachment from their environment. The parents’ perception of their child’s ability was expressed in the use of multiple images of objects and in concrete titles for the artworks. The complexity of family relationships and the tension created by the conflicts that exist beneath the surface were expressed in the families’ difficulty to converge around a single family image despite the instructions, the low percentage of images representing conflict, and the minimal presence of faces which has been linked in the literature to existing unexpressed conflict. Finally, the need for structure and control was also seen in the accentuation of the outline of the images.

Nevertheless, the drawings also generated signs of hope and optimism. The presence of moderate colorfulness shows the potential for development and growth. It is important to reiterate that for most of the families who participated in the study, the style of communication was verbal. It is possible that communication in sign language would have enabled the existing and observable potential of the drawings to be expressed in words and as such the relationship between the child and his family could have been better clarified.

The findings thus demonstrate how the use of the Family Squiggle Drawing Technique to assess the family climate of hearing families with a D/HH child enabled these families to somewhat bypass language and communication barriers and as such contributes to a broader understanding of the family climate and dynamics. Using this tool, both clinically and in research can facilitate a deeper understanding of hearing families with D/HH children.

### Research Limitations and Suggestions for Future Research

This study constitutes the first exploration of the family climate of families with a D/HH child during which both parents and the child simultaneously participated in joint artwork. It is also the first time squiggles have been applied to assess the family climate of families with a D/HH child using a joint drawing technique. Thus, the findings derived from this preliminary work should be considered with caution. The study was conducted in the families’ homes, which allowed the researchers to encounter the families in their natural environment but also conveyed a sense of affinity and caring for families who often need to consult professionals in medical settings. However, the impact of factors such as social desire or a sense of commitment on the findings should be considered. In addition, we did not distinguish between degrees of hearing loss and communication style, which could affect the relationships and family climate. While this sample enabled us to access data from as many families as possible, it was more difficult to examine the impact of these factors on the family climate. Finally, the analysis was conducted on the family level and did not explore the contribution of individual family members in the family climate. Further research should explore the family climate with a larger sample while taking these features into account.

Overall, the present study described an assessment tool of pictorial phenomena that characterize hearing families who have a D/HH child. Future research could consider applying this tool when working with families of children with other special needs to better understand the conscious and unconscious facets of children’s relationship with their parents.

## Data Availability Statement

The raw data supporting the conclusions of this article will be made available by the authors, without undue reservation.

## Ethics Statement

The studies involving human participants were reviewed and approved by The Chief Scientist of the Ministry of Education, Israel. Written informed consent to participate in this study was provided by the participants’ legal guardian/next of kin.

## Author Contributions

This research was part of a Ph.D. dissertation, which was initiated, planned, coordinated, and conducted by AA-W. She collected the data, carried out the analyses, and wrote the manuscript. DR and SR supervised the Ph.D. dissertation and assisted in all stages. All authors contributed to the article and approved the submitted version.

### Conflict of Interest

The authors declare that this study was conducted in the absence of any commercial or financial relationships that could be construed as a potential conflict of interest.
